# Latent Class Analysis of School Refusal Behavior and Its Relationship With Cyberbullying During Adolescence

**DOI:** 10.3389/fpsyg.2019.01916

**Published:** 2019-08-16

**Authors:** B. Delgado, M. C. Martinez-Monteagudo, C. Ruiz-Esteban, E. Rubio

**Affiliations:** ^1^Department of Developmental Psychology and Didactic, Faculty of Education, University of Alicante, Alicante, Spain; ^2^Department of Developmental Psychology and Education, Faculty of Psychology, University of Murcia, Murcia, Spain

**Keywords:** school refusal behavior, cyberbullying, cybervictimization, latent class analysis, adolescence

## Abstract

Cyberbullying is a common relational problem having negative repercussions on the academic performance of adolescents. Numerous questions remain to be answered with regard to the relationship between cyberbullying and school refusal behavior. This study examines school refusal profiles (measured by School Refusal Assessment Scale-Revised) and assesses whether these profiles vary with respect to the level of victimization, aggression, aggression-victimization, and observation of cyberbullying (measured with the Screening of Harassment among Peers). The sample consisted of 1,102 Spanish high school students, aged 12–18 (*M* = 14.30, SD = 1.71). Latent class analysis revealed three school refusal behavior profiles: non-school refusal behavior, school refusal behavior by negative reinforcements (oriented to the avoidance of social evaluation and negative affectivity in school situations), and school refusal behavior by positive reinforcements (oriented to obtaining the attention of others with significant or tangible reinforcements). The ANOVA found statistically significant differences for all cyberbullying behaviors. Students with school refusal by negative reinforcements had significantly higher mean scores as compared to the other profiles in victimization, aggression, aggression-victimization, and observation behaviors, while the levels of cyberbullying were similar between students without school refusal and students with school refusal behavior by positive reinforcements. These findings underscore the need to consider priority interventions to prevent cyberbullying in children who refuse school for the purpose of avoiding situations of anxiety and negative emotions.

## Introduction

School refusal behavior (SRB) is defined as the difficulty in attending or remaining in school for the entire day (Hendron and Kearney, 2011). This phenomenon has multiple causes and affects approximately 30% of all minors aged 7–17 (Mihalas, 2014; Organization for Economic Cooperation and Development, 2016). SRB includes all types of school absenteeism in which symptoms of anxiety may or may not exist, such as school rejection or truancy (Kearney and Albano, 2018). It is considered to be a significant educational and health problem, given its numerous negative consequences. Evidence from prior studies suggests that SRB is related to: (a) internalizing problems, such as comorbidity with anxiety problems due to separation, generalized anxiety, social anxiety, and oppositional defiant disorder; (b) externalizing problems, such as aggressive behavior, consumption of drugs and alcohol; and (c) health problems such as asthma, migraines, obesity, etc. (Kearney, 2008; Gonzálvez, 2018; for a review). In addition, chronic absenteeism may impair academic performance, being the most likely cause of early school drop-out, and placing minors at risk of developing problems such as drug consumption, delinquent behavior, social adjustment issues, and mental health problems (Dembo et al., 2013), thus leading to a decreased chance of attaining qualified and stable employment during adulthood (Wilson et al., 2008).

Therefore, it is important to understand the causes that may lead students to reject and not attend school as well as to determine the different profiles in these youth who do not attend school, in order to improve the prevention or intervention strategies employed in the scholastic context. Of the most relevant contemporary theoretical approaches, we find the functional model proposed by Kearney and Silverman (1993) which establishes a classification of SRB based on the school rejection motivation, including a large percentage of youth having school attendance issues. This model distinguishes between four functional conditions that underlie SRB: (1) avoidance of school-based stimuli that provoke negative affectivity (e.g., distress, anxiety, depression); (2) escape from aversive social and/or evaluative situations (e.g., tests, peer interactions); (3) pursuit of attention from significant others (e.g., parents); and/or (4) pursuit of tangible reinforcers outside of school (e.g., sleeping, watching television, playing video games). These conditions are grouped together, taking into account the behavioral consequences of the minors’ responses. So, the first two conditions refer to school refusal behavior based on negative reinforcement or the avoidance of aversive situations, whereas the latter two conditions refer to school refusal behavior that is based on positive reinforcement or obtaining something positive outside the school (Kearney, 2002). This functional classification system has considerable advantages, such as a greater ability to distinguish between the different causes of SRB with and without anxious symptomatology (Gonzálvez et al., 2018; Sanmartín et al., 2018), and therefore, an increased specification and efficiency in the implementation of intervention strategies for each student.

Based on the functional classification system, prior studies have attempted to analyze the SRB profiles in children and adolescents (Dube and Orpinas, 2009; Gonzálvez et al., 2018). For instance, Dube and Orpinas (2009) in a clinical sample of 99 US students aged 8–15 with school attendance problems detected three profiles: a profile of multiple SRB having negative and positive reinforcement factors (17.2%), another SRB profile to obtain tangible positive reinforcement or parent’s attention (60.6%), and another non-SRB (22.2%). In addition, students with multiple SRB had significantly more behavioral problems (emotional problems, behavior problems, hyperactivity, and social problems with peers), and a higher frequency of victimization, aggression, and traumatic or stressful events. Gonzálvez et al. (2018), in an analysis of conglomerates based on a community sample of 1,582 Colombian students aged 12–18, found three distinct profiles: a group that did not reject school (44.8%), another that rejected school to obtain tangible reinforcers (42.9%), and a third group that rejected school for distinct motives such as to avoid situations causing negative emotions or social assessment and to attract the attention of significant others, such as their parents (12.2%). They also found that the group having the worst psycho-social adjustment with higher levels of anxiety, depression, and stress was the group that rejected going to school due to distinct causes (Gonzálvez et al., 2018).

In addition to low psycho-emotional and academic adjustment, SRB has also been strongly associated with variables of social interaction. So, many authors have suggested that poor relationships with schoolmates and bullying are significant factors in determining school rejection and absenteeism (Dube and Orpinas, 2009; Barboza, 2015; Havik et al., 2015). Barboza (2015) found that being victimized was related with an increased risk of developing escape and avoidance responses in the school environment, as well as skipping class and staying home during school hours. Havik et al. (2015), using a structural equations model found that being a victim of bullying was related to school rejection. They also found that social isolation and a lack of friends had more negative repercussions on minors who reject school since they caused negative emotions, whereas those of absenteeism/truancy had a lower impact since the students could be popular in school while maintaining social friendship networks outside of the school setting.

However, the phenomenon of victimization and bullying between peers is not unique to the school setting. With the widespread and generalized use of the information and communication technologies and the social networks, minors today are immersed in an environment in which they are more likely to suffer from or perpetrate these acts of bullying, better known as “cyberbullying” or “electronic bullying.” Cyberbullying has been defined as an aggressive action carried out repeatedly and deliberately through electronic means, toward an individual who cannot easily defend him/herself (Smith and Steffgen, 2013). The main roles involved in cyberbullying are: the victims, or those who suffer victimization; the aggressors, or people who perpetrate the harassment; and the observers, or individuals who witness the cyberbullying behaviors but do not directly take part in them. Another role has also been identified which includes people who, being victims, develop online bullying behaviors, and it is called the bully-victims (Schultze-Krumbholz et al., 2018). The prevalence of cyberbullying varies depending on the study (10–40%) and its negative consequences on psychological and social adjustment of the minors are multiple (Kowalski et al., 2014; Morin et al., 2018; for a review).

On account of cyberbullying research, it is important to mention that there is a lack in its theoretical foundation. In this sense, it is common to apply the general aggression model and the socio-ecological model to explain the potential influence of contextual and personal factors as risk elements for the development of harassment situations and aggression (Morin et al., 2018). Thus, among the personal variables, emotional problems and the perception of threat and insecurity in the educational context have been identified as risk variables for refusing school and being absent, while among the contextual factors, the disorganization of schools in matters of respect and violence control have been associated with greater absenteeism (Kearney, 2008). Thus, as occurs with face-to-face bullying, cyberbullied students tend to have a greater likelihood of being absentees (Barboza, 2015; Steiner and Rasberry, 2015; Grinshteyn and Yang, 2017). Barboza (2015), in a sample of 5,589 US adolescents, found that cyberbullying was related to escape behaviors in the school context, unjustified absences, and staying at home during school hours. Grinshteyn and Yang (2017), with a sample of 13,554 US students aged 14–18, found that the cyberbullied students were at a greater risk of being absentees as compared to those adolescents who were not victims of said cyberbullying. In addition, students who had experienced situations with violence, who had been threatened, or who felt sad or useless during the past year, also had higher probabilities of not attending school. Steiner and Rasberry (2015), analyzing 13,583 high school students, in grades 9–12, found that, with regard to the relationship between absenteeism and victimization (in person and electronic), minors who were victims of traditional bullying and cyberbullying were more likely to be absent from school since they considered it to be an unsafe place. Specifically, female victims of cyberbullying were 2.10 times more likely to not attend class, whereas this increased to a risk of 5.34 times, when they were victimized both *via* internet and in person. Male victims of cyberbullying were 3.58 times more likely to be absentee students, with this risk increasing to 6.68 if they were victimized both in the traditional manner and *via* electronic means (Steiner and Rasberry, 2015).

On the other hand, some studies have related the level of absenteeism and school rejection with being an aggressor or perpetrator of cyberbullying (Wright, 2015; Morin et al., 2018). A longitudinal study extending over 1 year found that in 673 US eight graders, perpetration and victimization *via* cyberbullying were both related to increased absenteeism and poorer academic performance after controlling for the prior level of absenteeism, and in-person bullying between peers (Wright, 2015). In addition, Morin et al. (2018), in a sample of 28,583 US high school students (grades 9–12), found that being a victim or aggressor of cyberbullying was associated with an increased risk of psychological issues such as internalizing problems, sleep disorders, and stress problems, as well as academic adjustment problems such as absenteeism (truancy) and poor academic performance. Specifically, the perpetrators of cyberbullying were 123.1% more likely to miss classes twice or more times per month (Morin et al., 2018).

Although it is relevant that silencing the aggression contributes to the perpetuation of harassment over time, no study to date has examined the relationship between SRB and cyberbullying observers. Cyberbullying observers or bystanders are a heterogeneous group composed by individuals who witness cyberbullying behaviors but do not involve in them directly (Schultze-Krumbholz et al., 2018). Bystanders can manifest negative consequences in their psycho-emotional adjustment (Garaigordobil, 2011; Wright, 2019), these include inferiority feelings, impotence, sadness, rage, guilt, and fear. If these emotional consequences are related with the educational context, this fact can lead them to refuse the school because it is perceived as an insecure environment (Grinshteyn and Yang, 2017).

This evidence suggests the importance of considering the negative consequences of cyberbullying on the academic adjustment of adolescents based on its direct implication on SRB. However, these studies have not considered the causes of the absenteeism and the functional analysis of SRB in terms of its relationship with cyberbullying. Furthermore, prior studies have considered the role of the victim and have, at times, considered the aggressor role (Wright, 2015; Morin et al., 2018) in explaining absenteeism, but they have not looked at other potentially important roles in cyberbullying such as that of the aggressor-victimized or the cyberbullying observer. It is necessary to determine the causes leading a minor to stop attending school and whether or not the distinct profiles of students who reject school may be related differently to the main roles of cyberbullying. This analysis provides keys that may help to establish better preventive measures and intervention strategies for the distinct groups of absentee students and those involved in the cases of cyberbullying. Furthermore, this study uses a classification process that is based on a latent variable mixture which surpasses the traditional statistical techniques (Schreiber, 2017).

The first objective of this study is to use latent class analysis to analyze the SRB profiles while considering the potential motives behind student rejection of school, based on a functional classification system. Taking prior studies into account (Dube and Orpinas, 2009; Gonzálvez et al., 2018), three SRB profiles are anticipated (one with low school rejection, another with rejection by positive reinforcement, and another with multiple causes for rejection). The second objective consists of examining the differences in cyberbullying (victimization, aggression, observation, and aggression-victimization) through the distinct SRB profiles that were previously determined. Taking into account these results, it is expected that adolescents with a high SRB profile will have higher scores on the cyberbullying roles than those with a low SRB profile.

## Materials and Methods

### Ethics Statement

All of the standards for research conducted with humans were respected according to the ethical principles of the Declaration of Helsinki (World Medical Association, 2013) and were guaranteed by the Ethics Committee of the Universidad de Alicante (Reference number: UA-2018-02-21).

### Participants

Participants were students from secondary-level education of the Valencian Community (Spain) during the 2017–2018 academic years. The Valencian Community approximately served a total of 261,000 secondary education students (Ministry of Education and Vocational Training, 2018). Two-stage random sampling was conducted. In the first stage, eight public and two charter secondary schools were randomly selected in Alicante province. Once the schools were selected, in the second stage of sampling, four classes were randomly selected from each school. Due to the random sampling method, the socioeconomic status and ethnic composition of the overall sample are assumed to be representative of the community. The study sample included 1,148 students, of which 46 (3.8%) were eliminated due to errors or omissions in their responses or because they did not obtain parental consent to participate in the study. The final sample consisted of 1,102 high school students, aged 12–18 (*M* = 14.30; SD = 1.71), with 509 males (46.2%) and 593 females (53.8%) participating. The sample’s distribution based on academic year was as follows: 184 (7th grade), 193 (8th grade), 190 (9th grade), 182 (10th grade), 208 (11th grade), and 145 (12th grade). The χ^2^ test was used to analyze the homogeneity of the sample in terms of gender and course, with no statistically significant differences being found between the groups of Gender x Course (*χ*^2^ = 2.97, *p* = 0.704).

### Measures

#### School Refusal Assessment Scale-Revised

The School Refusal Assessment Scale-Revised (SRAS-R) is one of the most widely-used questionnaires for the measurement of SRB considering functional conditions (Kearney, 2002; adaptation of Gonzálvez et al., 2016). The questionnaire consists of 24 items that are responded to using a 7-point Likert scale (0: *never;* 6: *always*) and that assess the relative self-perception of the four fundamental factors of SRB: *avoidance of school situations that provoke negative affectivity* (ANE; e.g., “How many times have you tried to avoid going to school because if you went you would feel sad or depressed?”), *escape from aversive social o evaluative situations* (ESE; e.g., “How many times have you tried to avoid going to school because it would be hard to talk to other boys/girls in the school?”), *pursuit of attention from significant others* (PA; e.g., “How many times would you have preferred to be with your family instead of going to school?”), and *pursuit of tangible reinforcement* (PTR; e.g., “How many times have you not gone to school because you wanted to have fun outside of school?”). The scale can be used for students from 8 to 17 years of age. SRAS-R scores have been found to have suitable psychometric properties in adolescents from distinct cultures (Richards and Hadwin, 2011; Seçer, 2014; Walter et al., 2017) and factorial invariance based on sex and age in Spanish school-aged populations (Gonzálvez et al., 2016) and in Chilean adolescent populations (Gonzálvez et al., 2018). In this study, the subscales of the questionnaire demonstrated an adequate reliability based on the Cronbach’s alpha values which were 0.77 for ANE, 0.75 for ESE, 0.80 for PA, and 0.78 for PTR.

#### Screening of Harassment Among Peers

The Screening of Harassment Among Peers (SPH) is a self-reporting instrument that assesses bullying and cyberbullying behavior in adolescents and youth taking place over the past year, *via* four subscales: *victimization* (behavior suffered by the bullying victim), *aggression* (bullying behavior perpetrated by the aggressor), *observation* (bullying behavior witnessed by the observer), and *aggression-victimization* (bullying behaviors that are suffered as a victim and perpetrated as an aggressor) (Garaigordobil, 2013). The questionnaire assessed 15 cyberbullying behaviors such as password and identity theft, anonymous calling to frighten, slander/spread rumors to discredit, send offensive/insulting messages, the dissemination of recorded aggressions or private videos over the Internet, the sexual bullying of others over the Internet, threats made so that secrets are not revealed over the network, and death threats made over the Internet. The cyberbullying questionnaire contains a total of 45 items and a Likert-like response format with four options (1: *Never*; 4: *Always*). The reliability of the instrument has been confirmed by the original authors in samples of Spanish adolescents (Garaigordobil, 2013, 2015). In this study, the internal consistency coefficients (Cronbach’s alpha) were satisfactory for the total score of the questionnaire (0.98) and for the subscales of victimization (0.95), aggression (0.96), observation (0.94), and aggression-victimization (0.98).

### Procedure

Initially, the researchers interviewed the management team of the selected schools in order to explain the purpose of the study. Then, an informative letter was sent to the parents of the minors in order to explain the study and to request their informed consent in writing. Questionnaires were responded to collectively and voluntarily in the classrooms during a class session, ensuring the anonymity of the participants and the confidentiality of the data. To do so, identification numbers were assigned on the response sheets of each participant. The researchers were present during the administration of the tests to clarify any potential doubts and to verify the correct completion of the questionnaires, which had a mean completion time of 15 min.

### Statistical Analyses

The SRB profiles were defined based on the combinational differences of the four functional conditions of the SARS-R, and were established using the latent class analysis (LCA). LCA is considered to be the most appropriate procedure for establishing profiles in large samples and it surpasses the limitations found in other statistical techniques such as the analysis of conglomerates (Schreiber, 2017). Considering the number of classes proposed by the researchers, subjects were included in one of the classes according to their profile. To select the number of classes that best represented the research data, the lowest indicator of the Bayesian Information Criteria (BIC) and the Akaike Information Criterion (AIC) were used as adjustment indices, as well as the value closes to one for the Entropy (Schreiber, 2017; Smeets et al., 2017). Finally, to calculate the differences for cyberbullying (victimization, aggression, observation, and aggression-victimization) between the distinct classes of SRB, ANOVAs were conducted as well as *post hoc* Scheffé tests to determine the groups between which there were statistically significant differences. Finally, the *d* index (standardized mean difference) proposed by Cohen (1988) was calculated, allowing for the assessment of the magnitude or effect size of the differences that were found. Its interpretation is simple: 0.20 ≤ *d* ≤ 0.50 means a small effect size, while 0.51 ≤ *d* ≤ 0.79 is moderate, and *d* ≥ 0.80 is large.

## Results

### School Refusal Behavior Profiles

**Figure 1:**
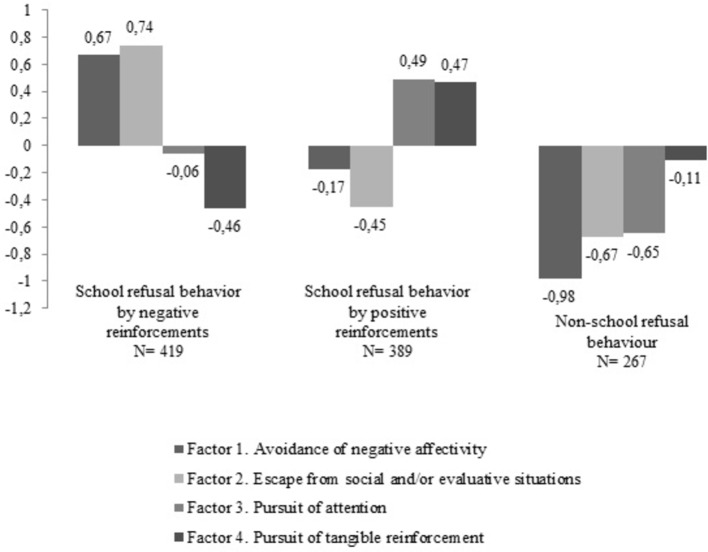
Graphic representation of the LCA solution. Note: SRB, School Refusal Behavior.

The LCA found that the class made up of three profiles with different levels of SRB, considering the four dimensions of the SRAS-R, ANE, ESE, PA, and PTR (see Figure 1), had the best adjustment for the BIC, AIC, and Entropy indicators (see Table 1). The first profile, SRB by negative reinforcements, included 419 students (38.02%) with high levels of ANE and of ESE and low levels of PA and PTR. The second profile, SRB by positive reinforcements, classified at 389 (35.29%) with high levels of PA and PTR and low levels of ANE and ESE. The third profile, non-SRB included 267 (24.22%) students having low scores on the four analyzed dimensions.

**Table 1:** Fit indices of the latent class analysis (LCA) values in bold revealing the best model fit.

No. of classes	BIC	AIC	Entropy	Number of parameters
2	11141.88	11057.22	0.767	17
**3**	**10127.93**	**9863.988**	**0.800**	**26**
4	10307.81	10088.68	0.750	35
5	10493.83	10319.53	0.767	44
6	10760.03	10630.54	0.742	53

*BIC, Bayesian Information Criterion; AIC, Akaike Information Criterion*.

### Inter-group Differences in Cyberbullying Behavior

The ANOVA found statistically significant differences between the SRB profiles for all of the cyberbullying roles. The results obtained from the *post hoc* tests indicate that the students with a SRB by negative reinforcements profile received significantly higher scores on victimization, aggression, aggression-victimization, and observation of cyberbullying than the non-SRB group and the group of students with SRB by positive reinforcement (see Table 2). However, these differences were not found between the profiles of SRB by positive reinforcements and the non-SRB students.

**Table 2:** Means and standard deviations of cyberbullying between classes and statistical significance.

	Negative reinforcement SRB		Positive reinforcement SRB		Non-SRB		*F*	*p*	*η*^2^
	*M*	SD	*M*	SD	*M*	SD			
Victimization	26.06	10.78	21.51	9.12	20.07	8.64	37.543	0.00	0.065
Aggression	25.20	10.97	20.61	9.48	19.23	8.65	36.088	0.00	0.063
Aggression-victimization	51.25	21.40	42.12	18.30	39.30	17.01	38.039	0.00	0.066
Observation	26.70	10.06	23.23	9.51	21.88	9.47	23.419	0.00	0.042

*SRB, school refusal behavior*.

As shown in Table 3, the effect sizes (mean standardized difference) for the differences found in cyberbullying were small in size for the groups of SRB by positive reinforcements and SRB by negative reinforcements (*d* < 0.46), whereas the differences between the group of SRB by negative reinforcement and the non-SRB were moderated by the differences in victimization, aggression, and aggression-victimization, and were small for the difference in observation of cyberbullying.

**Table 3:** Cohen’s *d* index to *post hoc* contrast between the means scores and the three classes in the roles of cyberbullying.

	Negative reinforcement SRB vs. positive reinforcement SRB	Negative reinforcement SRB vs. non-SRB	Positive reinforcement SRB vs. non-SRB
Victimization	0.45	0.60	n.s.
Aggression	0.44	0.59	n.s.
Aggression-victimization	0.46	0.60	n.s.
Observation	0.35	0.49	n.s.

*SRB, school refusal behavior; n.s., non-significant differences*.

## Discussion

This study had two objectives: first, the analysis, *via* latent class analysis, of the SRB profiles, taking into account the motives leading students to reject school, according to the four-factor functional model (Kearney and Silverman, 1993), and second, to examine the differences in cyberbullying (victimization, aggression, observation, and aggression-victimization) through the different SRB profiles in a sample of Spanish high school students.

In line with the results obtained from prior studies (Dube and Orpinas, 2009; Gonzálvez et al., 2018), three SRB profiles were anticipated: one with low school rejection, another with rejection by positive reinforcement, and another with multiple causes for the rejection. The results of the study suggest the existence of three SRB profiles but they differ slightly from the expected results. A class of students was found that rejected school in order to avoid negative emotions and stressful social and assessment situations (38%; SRB by negative reinforcements). This profile did not coincide with that found in prior studies, since the negative reinforcement factors of school rejection (avoiding or escaping negative situations and emotions) were not grouped together with positive reinforcement factors (obtaining parents’ attention) in a group or profile of multiple school rejection (Dube and Orpinas, 2009; Gonzálvez et al., 2018). Also, in this work, the incidence of students rejecting school due to an emotional or anxiety-based component is higher than the group that rejected school due to a variety of causes (12.2–17.2%). These findings, while dissenting, reinforce the contributions of Kearney (2002) and Kearney and Albano (2004) which combined the dimensions of ANE and ESE since they were considered to be similar.

On the other hand, a second class was identified which was characterized by students who rejected going to school in an attempt to obtain their parents’ attention and other tangible reinforcers outside of the school (35.3%; SRB by positive reinforcements). This class coincides with the findings of Dube and Orpinas (2009) and coincides partially with those of Gonzálvez et al. (2018) in the conglomerate of rejection by tangible reinforcements; however, it differed in the quantity of students grouped in this class, since the prevalence is lower than in prior studies (42.9–60.6%). This may be due to differences in age and community characteristics of the sample examined in this study. Finally, the prevalence of students who did not reject school (24.22%; non-SRB) was similar to that found for students with school attendance problems (Dube and Orpinas, 2009) (22.2%) and lower than that found in the Colombian adolescents (Gonzálvez et al., 2018) (44.8%). Therefore, the initial study hypothesis can only be partially confirmed.

Given the results for the three latent classes, the cyberbullying behavior was analyzed, finding inter-class differences in the scores for victimization, aggression, observation, and aggression-victimization. The profile of students with SRB by negative reinforcements had significantly higher scores than the other profiles for all cyberbullying behaviors. These results confirm the second hypothesis which anticipates that the adolescents with a high SRB profile would have higher scores in cyberbullying and reinforces the findings of prior studies that found cybervictimized adolescents to be a population at risk of not attending school because they did not feel safe (Steiner and Rasberry, 2015; Grinshteyn and Yang, 2017) and in order to engage in escape and avoidance behavior in an educational context (Barboza, 2015). Furthermore, the results are in line with those from other studies with adolescents that have related the level of absenteeism and school rejection with being an aggressor or perpetrator of cyberbullying (Wright, 2015; Morin et al., 2018), and high aggression levels with higher levels of school rejection in order to avoid negative affectivity and social evaluation and to gain the attention of significant others and similar levels of school rejection to obtain tangible reinforcers (Vicent et al., 2018).

However, students with SRB by positive reinforcements are not different from the non-SRB group in terms of the four cyberbullying roles. This may be explained by the different impact of cyberbullying according to the school rejection declarations. Havik et al. (2015) found that victimization, social isolation, and a lack of friends may have more negative repercussions on minors who reject school since they lead to negative emotions, whereas for those having a non-anxious/truancy rejection profile (e.g., to obtain tangible reinforcements), the impact is less intense, since they may be popular in the school in addition to maintaining social friendship networks outside of the school setting. So, the authors conclude that anti-bullying actions should be mainly directed toward those students who reject school in order to prevent negative emotions, as opposed to absentee students who seek to obtain tangible reinforcement by skipping class (Havik et al., 2015). Furthermore, students with SRB by positive reinforcements are found to have greater emotional adjustment, which may result in an improved ability to handle cyberbullying situations. Thus, Gonzálvez et al. (2016) found that school rejection that was intended to decrease negative emotions and social situations was more closely related to negative and pessimistic emotions, whereas this relationship was not found in those students who skipped school in order to obtain tangible reinforcement, who were shown to have higher levels of positive emotions and optimism and lower levels of pessimism.

The results of this work expand upon the results of prior studies, analyzing other important roles in cyberbullying such as that of the aggressor-victimized and the cyberbullying observer. This study found that students with SRB derived from a high negative emotionality and avoidance of evaluation and social situations had higher scores on aggression-victimization and on the observation of cyberbullying behaviors. Like in other studies (e.g., Schultze-Krumbholz et al., 2018) in which bully-victims are identified as less socially competent, with high levels of aggression and low levels of empathy, the results of this study underline that SRB by emotional or social problems may manifest more aggression-victimization behaviors than truancy adolescents or those who do not reject school. In addition, students who reject school by emotional and social problems have less social skills and can use technologies as a measure of socialization with their peers, which can lead them to observe or suffer more cases of cyberbullying (Marques et al., 2018). Moreover, it is common that cyber aggressors are classmates or schoolmates (Karna et al., 2010; Festl et al., 2013); so, students with more emotional and social difficulties would avoid going to school in order to not to meet face-to-face with their aggressors and trying to reduce the fear or anxiety they feel. Therefore, it was assumed that the group of students with SRB by negative reinforcements is related with committing and suffering cyberbullying actions and of observing them, as with the traditionally analyzed cyberbullying roles (victim and perpetrator). These findings once again highlight the need to consider that the cyberbullying experiences in adolescents may lead to unjustified school absences due to the associated increase in fear, discomfort, and anxiety (Steiner and Rasberry, 2015; Grinshteyn and Yang, 2017), and that this situation, if extending over time, may have a negative impact on school adjustment, leading to poor academic performance, as is the case with in-person bullying between peers (Barboza, 2015; Morin et al., 2018).

### Limitations and Practical Implications

This study has certain limitations, including the impossibility of generalizing the results to other education levels and to other countries. Future studies should analyze whether or not the findings differ in other academic levels and in other cultures. Furthermore, the cross-sectional design used in the study makes it impossible to establish causal relationships. Therefore, it is recommended that longitudinal studies be carried out to provide additional information on the evolution of the SRB phenomenon and cyberbullying over the years. In addition, regarding Schultze-Krumbholz et al. (2018), cyberbullying observers or bystanders can be involved in an active way (either encouraging the bully to continue with the abuse or helping the victim to get out of the situation) or a passive way (looking the other way and allowing the harassment). These two differentiated characteristics of behavior should be evaluated in future research to assess their relation with the SRB. Finally, it should be noted that the assessment of the constructs has only been carried out using self-reports; therefore, it may be useful for future studies to consider multi-source (e.g., parents, teachers, counselors) and multi-methods assessments (e.g., interviews, questionnaires, observation, self-recording). Despite these limitations, this study provides some novel and important information for the study and understanding of SRB and their relationship with cyberbullying during adolescence, since it focuses on the functional characteristics of SRB and its relationship with all of the roles of cyberbullying, thus permitting the creation of defined profiles that facilitate the understanding of the phenomenon and an improved efficacy of the preventive strategies.

To conclude, this study has found the existence of three profiles of adolescents who reject school, with the most prevalent profile (38.02%) having a negative emotional component whose motives for rejecting school include avoiding negative emotions and social and assessment situations in the school (SRB by negative reinforcements). These students also correspond with the profile of having a higher level of cyberbullying behavior, both as victims and aggressors, aggressor-victim, and observer. Thus, strategies to prevent cyberbullying in academic settings should focus on the identification and intervention of cases, taking SRB into account, especially in adolescents who reject school to avoid situations of anxiety and/or negative emotions.

## Data Availability

The datasets generated for this study are available on request to the corresponding author.

## Ethics Statement

The studies involving human participants were reviewed and approved by the University of Alicante. Written informed consent to participate in this study was provided by the participants’ legal guardian/next of kin.

## Author Contributions

BD conceived the study and participated in its design, coordination, statistical analysis, and the manuscript drafting. MM-M participated in the study design and data interpretation, while also assisting in the drafting of the manuscript. CR-E performed a critical review of the manuscript and assisted with interpretation of the findings. ER assisted with the study conception. All authors read and approved the final manuscript.

### Conflict of Interest Statement

The authors declare that the research was conducted in the absence of any commercial or financial relationships that could be construed as a potential conflict of interest.
